# Implementation of Therapeutic Virtual Reality Into Psychiatric Care: Clinicians' and Service Managers' Perspectives

**DOI:** 10.3389/fpsyt.2021.791123

**Published:** 2022-01-04

**Authors:** Olivia S. Chung, Tracy Robinson, Alisha M. Johnson, Nathan L. Dowling, Chee H. Ng, Murat Yücel, Rebecca A. Segrave

**Affiliations:** ^1^BrainPark, Turner Institute for Brain and Mental Health and Monash Biomedical Imaging Facility, Monash University, Melbourne, VIC, Australia; ^2^School of Nursing, Paramedicine and Healthcare Sciences, Charles Sturt University, Bathurst, NSW, Australia; ^3^Professorial Unit, Department of Psychiatry, The Melbourne Clinic, The University of Melbourne, Melbourne, VIC, Australia

**Keywords:** implementation, virtual reality, barriers, facilitators, qualitative study, mental health, psychiatry

## Abstract

**Objectives:** Virtual reality (VR) has emerged as a highly promising tool for assessing and treating a range of mental illnesses. However, little is known about the perspectives of key stakeholders in mental healthcare, whose support will be critical for its successful implementation into routine clinical practise. This study aimed to explore the perspectives of staff working in the private mental health sector around the use of therapeutic VR, including potential implementation barriers and facilitators.

**Methods:** Semi-structured qualitative interviews were conducted with cross-disciplinary clinicians (*n* = 14*)* and service managers (*n* = 5), aged 28–70 years working in a major private mental health hospital in Victoria, Australia. Transcripts were analysed using general inductive coding to allow themes to naturally emerge.

**Results:** Three major themes were identified: clinical factors (four subthemes), organisational factors (five subthemes), and professional factors (three subthemes). The themes encompassed enabling factors and potential barriers that need to be addressed for successful implementation of VR. Clinical factors highlighted the influence of knowledge or perceptions about appropriate clinical applications, therapeutic efficacy, safety and ethical concerns, and patient engagement. Organisational factors emphasised the importance of service contexts, including having a strong business case, stakeholder planning, recruitment of local opinion leaders to champion change, and an understanding of resourcing challenges. Professional factors highlighted the need for education and training for staff, and the influence of staff attitudes towards technology and perceived usability of VR.

**Conclusions:** In addition to enabling factors, potential implementation barriers of therapeutic VR were identified, including resourcing constraints, safety and ethical concerns, negative staff attitudes towards technology and VR system limitations. Future dissemination should focus on addressing knowledge and skills gaps and attitudinal barriers through development of clinical guidelines, training programs, and implementation resources (e.g., adoption decision tools, consultation opportunities).

## Introduction

Virtual reality (VR) has emerged as an efficacious tool for treating a wide range of mental illnesses, and a highly promising method for assessing them (1, 2). VR is a computer-generated three-dimensional environment that enables immersive multi-sensory experiences, typically through using a head-mounted display, headphones and hand-held controllers. VR affords a sense of “presence” (i.e., “being there”) and embodiment of a virtual body, which can be exploited to enhance traditional psychological treatment by providing safe, ecologically valid virtual environments to modify maladaptive thoughts, behaviours and internal body representations (3, 4). Moreover, VR offers advantages in providing greater access and control over stimuli otherwise impractical or costly to replicate in clinical settings (2, 5). The largest clinical application has been VR exposure therapy (VRET) for anxiety-related disorders (e.g., phobias, social anxiety, agoraphobia, PTSD), with over 30 randomised control trials supporting its efficacy, with equivalent effect sizes (6) and attrition rates to *in vivo* exposure therapy (7, 8). Moreover, evidence supporting its efficacy across an increasingly broad range of conditions is growing, including autism-spectrum disorder, addictions, eating disorders, and psychosis (9, 10).

In 2016, the release of affordable VR platforms (e.g., HTC Vive, Oculus Quest) and expansion of application distribution channels (e.g., app stores) heralded an unprecedented opportunity for VR applications to be brought to mental healthcare at scale. Indeed, some have predicted that VR “will soon become standard tools in the toolbox” of clinicians (11). To this effect, the National Institute of Health Research (NIHR) awarded £4 million to support trialling of VR-based psychological therapies through the United Kingdom's publicly funded healthcare system (12). However, implementing VR into mainstream clinical settings will be challenging, as historically, only about half of new evidence-based practises reach widespread adoption, taking on average 17 years to become routinised (13). Moreover, this lag appears even greater for psychological treatments (14).

Currently, knowledge of factors that will impact VR implementation in mental healthcare remains extremely limited, with most of this research pre-dating the release of consumer headsets, which has substantially changed implementation considerations (15–18). Moreover, with the exception of Lindner et al. (18), these studies have focused narrowly on applications in exposure therapy, leaving knowledge about its wider implementation within general mental health services scant. Additionally, prior research has seldom explored perspectives of service managers and cross-disciplinary clinicians across the mental health workforce (e.g., psychologists, psychiatrists, mental health nurses, allied health), who are gatekeepers to VR adoption, and whose support will inherently determine whether VR is integrated into routine practise.

Given mounting efficacy evidence and early adoption efforts for VR interventions (19), it is timely to better understand the perspectives of mental health service providers to facilitate sustainable uptake (20). Focusing on private mental health sector will be strategic as they are frequently earlier adopters of new evidence-based practises, compared with the public sector (21). This study sought to explore the perspectives of clinicians and service managers working in private mental healthcare regarding VR use, including potential implementation barriers and facilitators.

## Methods

Study reporting is based on the Consolidated Criteria for Reporting Qualitative Research (COREQ; see Supplementary File S1) (22).

### Study Setting

The study was conducted at The Melbourne Clinic, a large private psychiatric hospital in Victoria, Australia, that provides general and specialised mental health services to adults aged 18 years and older. The hospital has 203 inpatient beds, as well as outreach and day program services, and provides services for over 3,000 patients annually, with the most commonly treated conditions including major affective disorder, eating disorders or obsessive-compulsive disorder (OCD), personality disorders, and alcohol misuse. The inpatient setting provides a range of targeted and specialised treatment programs for eating disorders, OCD, addictive behaviours, trauma, as well as psycho-geriatric and acute psychiatric disorders. The hospital offers biomedical (e.g., psychopharmacology, electroconvulsive therapy, transcranial magnetic stimulation) and psychological interventions (e.g., cognitive behavioural therapy, dialectical behavioural therapy, trauma-focused treatment).

The hospital employs 428 personnel, and is staffed by multidisciplinary teams comprising psychiatrists, psychologists, art therapists, dietitians, social workers, exercise physiologists, mental health-trained nurses, and occupational therapists. Regarding experience to virtual reality, the hospital was the site of an experimental study validating VR scenarios for use in obsessive-compulsive disorder (OCD) treatment (23). However, VR is not currently being used as a clinical treatment tool within the hospital, thus no participants are currently involved in its use.

### Participant Characteristics

Of the 17 clinicians invited, 14 (82%) participated, and of the six managers invited, five (83%) participated. Non-participation was due to workplace change, scheduling difficulties, or non-response. Participant characteristics are summarised in Table 1. Clinical staff were primarily from a nursing background (43%), female (71%), 39.93 (*SD* = 12.06) years old, reporting 9.92 (*SD* = 10.09) years of clinical experience on average. Clinical roles involved direct therapy delivery to patients individually or in therapy group, clinical trial support, and broader psychiatric care (e.g., medication management, intake assessment and triage). Managers were primarily female (80%), 44.80 (*SD* = 19.69) years old, reporting 5.35 (*SD* = 4.68) years of clinical or service management experience, with two participants holding a dual clinical role. None of the participants had used VR in a treatment setting, however, seven (42%) participants reported having previously experienced VR recreationally. All participants reported having heard of VR's therapeutic application (e.g., through study advertising, conferences, prior research participation). In the event VR were to be introduced within the hospital, nine participants may be likely to deliver VR-supported psychological therapies directly. The remaining five clinical staff may also be eligible to deliver VR depending on training and implementation support.

**Table 1:** Sample characteristics.

**Characteristics**	***N*** **(%) or mean (SD)**	
	**Clinical (*n* = 14)**	**Manager (*n* = 5)**
Clinical background		
Mental health nurse	6 (43)	
Psychologist	2 (14)	
Psychiatrist	2 (14)	
Other allied health^a^	4 (28)	
Settings worked in current role (multiple answers)		
Inpatient	7 (50)	
Outpatient (e.g., outreach, day program)	5 (35)	
Intake	2 (29)	
Clinical Research	2 (14)	
Years of clinical/management experience	10 (10)	5 (5)
≤ 1 y	1 (7)	1 (20)
2–5 y	5 (36)	2 (40)
6–10 y	3 (21)	1 (20)
11–15 y	2 (14)	1 (20)
16–20 y	1 (7)	
> 20 y	2 (14)	
Age (years)	40 (12)	45 (20)
20–29 y	3 (21)	
30–39 y	6 (43)	2 (40)
40–49 y	3 (21)	2 (40)
≥50 y	3 (21)	1 (20)
Gender		
Male	4 (29)	1 (20)
Female	10 (71)	4 (80)
Prior recreational use of virtual reality		
Yes	5 (36)	2 (40)
No	9 (64)	3 (60)

a *Other allied health: counsellor, occupational therapist, intake clinician*.

### Data Collection

To glean an in-depth understanding of mental healthcare staff's perspectives and preferences regarding VR, qualitative, single, semi-structured interviews (mean 37 min) were conducted in-person by O.C (58%) and A.J (42%) between June 2018 and April 2019. To be eligible, participants needed to be a current staff member of the hospital, with clinical or managerial experience. Purposive sampling was used to identify participants with diverse disciplinary backgrounds (including psychiatry, psychology, nursing, and allied health) and those who had experience in providing clinical care and managing operations of a private psychiatric service. Respondents from a previous survey study who had indicated their willingness to be involved in future interviews (Chung et al., in preparation), as well as staff identified by managers as potential informants who could provide rich information about potential applications, were invited to participate via email or phone. Sampling was further influenced by, and ceased, when thematic saturation was achieved. Respective interview schedules for clinicians and managers were pilot-tested and emailed to participants prior to interviews. Questions probed the *acceptability, appropriateness*, and *feasibility* of VR, as these have been identified by Proctor and colleagues (24) as critical constructs to understand for early implementation, alongside barriers and facilitators relevant to participants' roles and service. Additionally, managers were asked to reflect on implementation experiences and processes around organisational change. Interviewers, O.C (PhD candidate, BA Hons/BSci) and A.J (Student, BA Hons) were female and trained by T.R (Researcher, PhD), an experienced qualitative researcher. Interviewers had no prior relationships with participants. Interviews were audio recorded, transcribed verbatim by a professional transcription service, and reviewed for accuracy by the research team. Interviewers maintained field notes and regularly debriefed with co-authors, with discussions influenced by backgrounds in neuropsychology, psychology, nursing, implementation, and qualitative research. Transcripts were de-identified prior to entry into NVivo for analysis (version 12; QS International).

### VR Immersion

**Figure 1:**
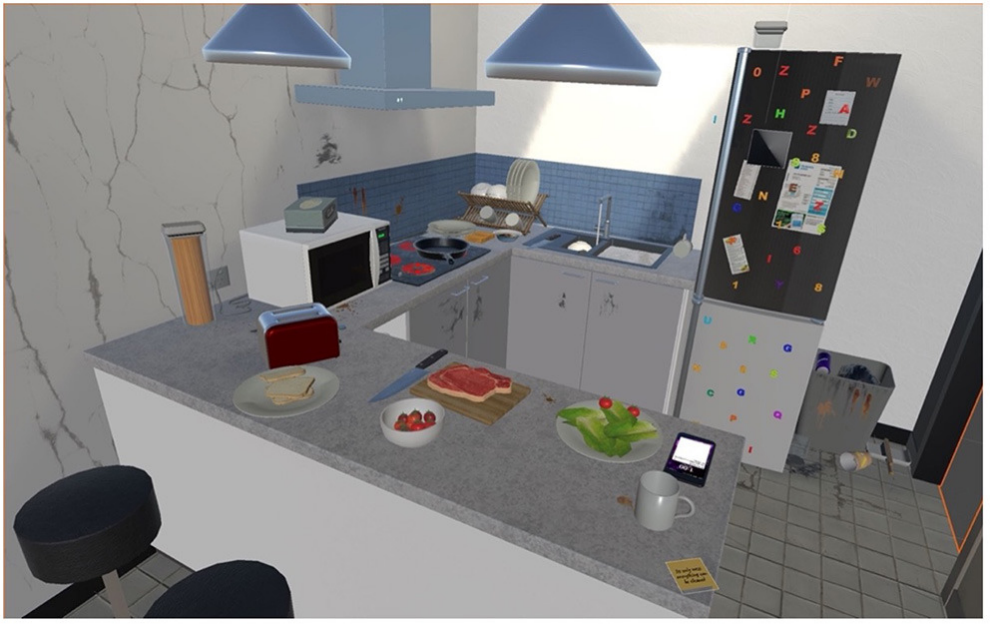
Example of VR scenario (i.e., domestic kitchen) developed for use in OCD treatment, experienced by participants during their interview immersion.

**Figure 2:**
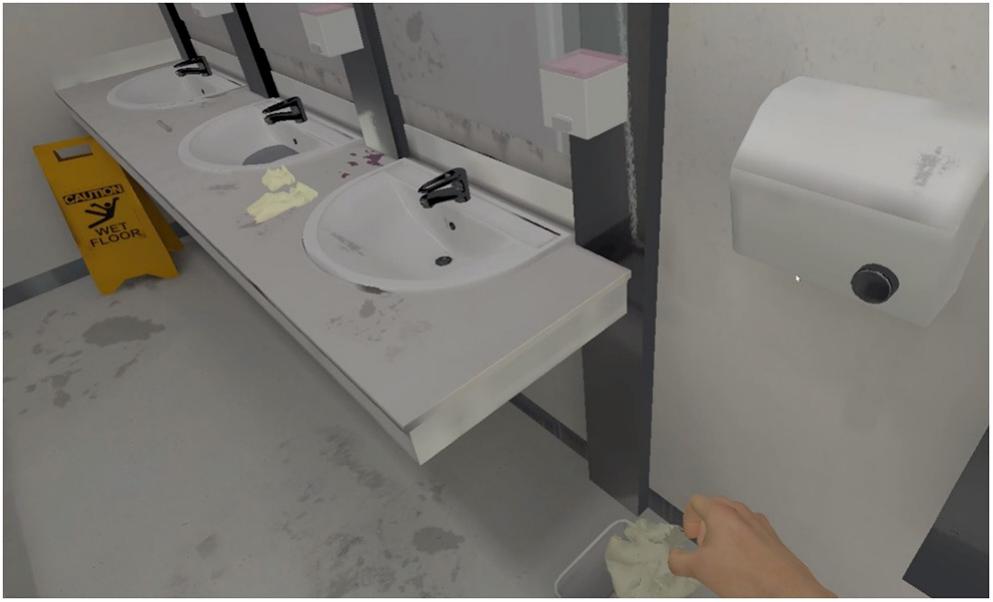
Example of VR scenario (i.e., public bathroom) developed for use in OCD treatment, experienced by participants during their interview immersion.

To maximise participants' knowledge and grounding of shared perspectives through a lived experience of therapeutic VR, all participants during their interview were immersed for 5–10-min in VR scenarios designed for OCD treatment (see Figures 1, 2) (25). A HTC Vive system with a wireless head-mounted display and handheld controllers was utilised.

### Data Analysis

Data was analysed using inductive content analysis that derives themes from the data by generating initial codes to identify specific topics that are subsequently compared, combined and analysed to develop broad themes (26). To ensure accuracy, transcripts were compared to their original audio files, and read and re-read by OC, who performed line-by-line coding, conceptualised the data and inductively identified similar concepts. A preliminary codebook was created by OC from identified concepts, which provided definitions, descriptions, qualifications, and examples utterances of codes, and which allowed the team to see and record how codes were being applied to the data (27, 28). Transcripts (15%) were co-coded against the codebook (OC, TR, RS) to enhance coding consistency and transparency around coding decisions. Coders met regularly to compare codes and interrogate themes against the study aim, helping to ensure confirmability of findings and reduce risk of thematic interpretation bias. The codebook was iteratively refined with input from co-coders, with subthemes and codes continuously added, revised, and arranged into meaningful groups until there was consensus regarding emerging primary and secondary themes. The codebook was considered final when a draft version of the codebook was applied to a larger data set and no new codes emerged. All transcripts were subsequently re-coded by OC against the final codebook. Links among themes were used to develop a thematic schema.

## Results

### Themes

**Figure 3:**
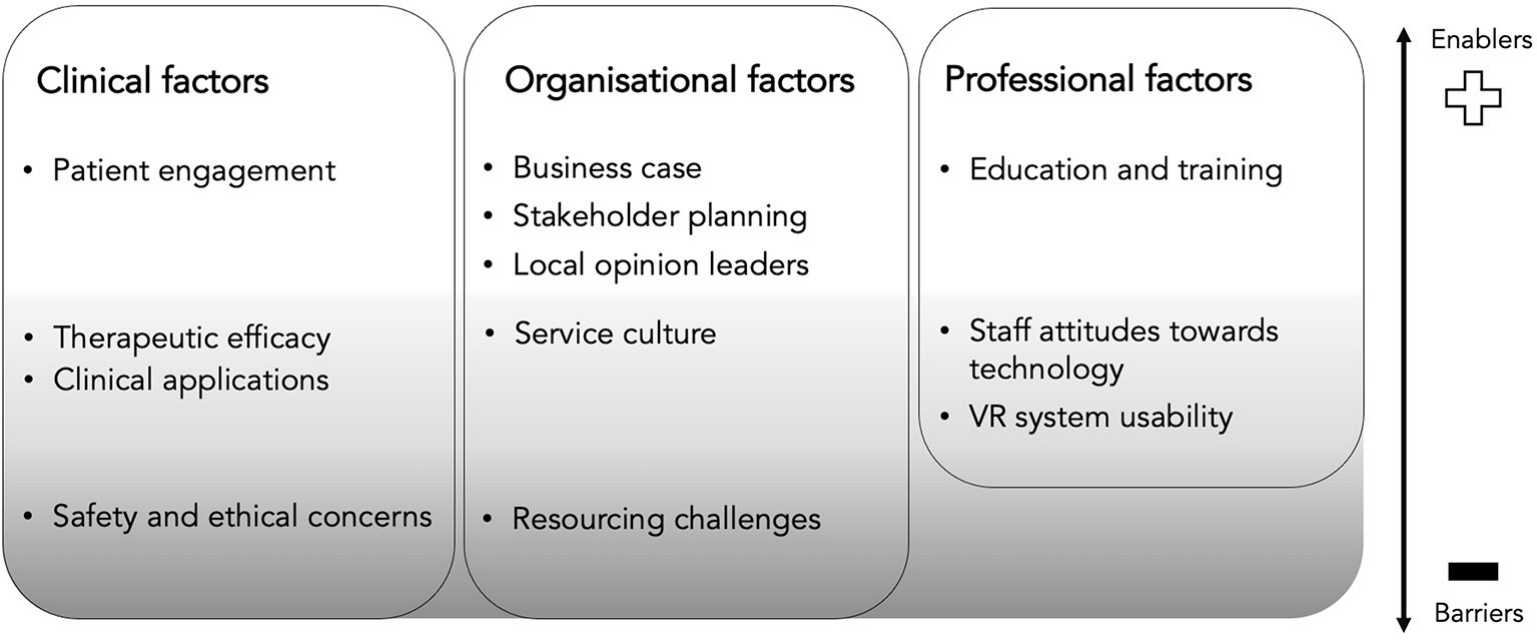
Thematic schema of clinicians' and managers' perspectives of therapeutic VR, including perceived implementation barriers and enablers. Barriers (dark grey) included safety and ethical concerns, and resourcing challenges. Factors representing mixed barriers and enablers depending on their presence or absence (light grey) include knowledge of and perceptions about clinical applications and their therapeutic efficacy, a service culture supportive of innovation, perceived usability of VR systems, and favourable staff attitudes towards technology. Enablers (white) include perceptions that VR will enhance patient engagement with treatment, having a strong business case, collaborative stakeholder planning, the use of local opinion leaders to champion change and provision of education and training to staff.

Thematic analysis revealed three broad themes: clinical factors (four subthemes), organisational factors (five subthemes), and professional factors (three subthemes). The themes encompassed enabling factors and potential barriers that need to be addressed for successful implementation of VR in clinical settings. The subthemes identify specific enabling factors relating to each theme, the absence of which comprise barriers to implementation (see Table 2 for definitions of themes and subthemes). Themes are schematically depicted in Figure 3. For a table of illustrative quotes, see Supplementary File S2.

**Table 2:** Definitions of themes and subthemes.

**Theme/subtheme**	**Definition**
*Clinical factors*	Clinical factors that may influence the perceived appropriateness of VR
Patient engagement	Perceived influence of VR on patient help-seeking or engagement with treatment
Therapeutic efficacy	Knowledge of, or questions regarding, therapeutic efficacy of VR-based therapies
Clinical applications	Knowledge of, or perceptions about, the appropriateness, relevance, or suitability of VR for a given clinical disorder (e.g., anxiety), setting (e.g., inpatient, outpatient), or intervention (e.g., exposure therapy).
Safety and ethical concerns	Concerns (actual or perceived) about identifying and/or managing ethical or safety risks (e.g., contraindications, side effects) when using VR with patients
*Organisational factors*	Service context factors that may influence the perceived appropriateness and feasibility of VR
Business case	A strong business case or rationale for implementing VR, which considers both benefits and costs/risks clinically and to the service
Collaborative stakeholder planning	Consultation and collaborative planning with key stakeholder groups (e.g., clinicians, managers, administrative staff, patients, private health funds)
Local opinion leaders	Recruiting and involving individuals with formal or informal influence over the attitudes/beliefs of their colleagues as a strategy to promote VR use
Service culture	Service cultural norms and values that may support or hinder the uptake of VR (e.g., patient-centred care, innovation)
Resourcing challenges	Perceived resourcing requirements and constraints of the service setting (e.g., cost, staffing, space) that may hinder VR uptake
*Professional factors*	Workforce-related factors that could influence the acceptability and feasibility of VR
Education and training	Availability and provision of promotional, educational, and training resources designed to increase familiarity with VR, develop skills and encourage use of VR clinically (e.g., workshops, seminars, guidelines, manuals)
Staff attitudes towards technology	Staff preferences and attitudes (positive, negative, neutral) towards technology, as influenced by their personal, professional, and organisational experiences
VR system usability	Perceived usability, complexity, or comfort of VR (e.g., hardware and software) based on past and current experiences with VR

### Clinical Factors

Participants identified a range of clinical factors likely to influence the perceived appropriateness of VR, that were grouped into four subthemes including: patient engagement, therapeutic efficacy, perceived clinical applications, and safety and ethical concerns.

#### Patient Engagement

Clinicians and managers (*n* = 17) were unanimous in perceiving VR as a tool that could “break down barriers” and enhance patient engagement with treatment. Staff felt VR's interactivity would benefit patients who found traditional therapy approaches difficult to engage with or sought alternatives to medication, which could encourage earlier or sustained help-seeking.

“*A new modality can be the one thing that might help you to break through with a patient if other things have failed, who might be stuck in a bit of a rut with just using verbal talking therapies… for people that might be more visually orientated.”* (P12, Psychiatrist)

Participants expressed that VR would have particular appeal for young people, who tended to be comfortable with technology:

“*With the younger generation, technology is a given now. They live in a virtual world by default most of the time. It could be a segue into that, into their world of interaction… Here's a situation where you've got something they can buy into, interact with. It could be a nice way of introducing concepts to them, therapeutic concepts.”* (P13, Nurse)

In relation to the engagement of older adults with VR, participants reported mixed views with some noting that older people would be more reluctant to use VR, however this response was not uniform. For instance, one clinician noted that:

“*Video games, Wii Fit, that sort of stuff with older populations has been really successful [in falls prevention]. Once it's tried out, I don't think it would be a resistant population group just because they don't know what it is.”* (P07, Occupational Therapist)

#### Therapeutic Efficacy

Another clinical factor identified as important to enable the implementation of VR was evidence of its therapeutic efficacy. Staff (*n* = 13) highlighted the need to be able to demonstrate the efficacy of VR to ensure its uptake, as it would be competing against more established therapies. Most staff were unaware of the current VR evidence-base, with five clinicians questioning whether VR would be experienced as “real enough” or as effective as current therapies:

“*I guess the questions around VR is whether it would translate to the same kind of clinical results… as it would in vivo [exposure]… Something tells me that in vivo would probably increase anxiety levels more so than the VR, which would mean that it would probably have better outcomes.”* (P04, Psychologist)

Thus, dissemination of efficacy evidence and having a clear evaluation plan for any implementation were identified as crucial clinical factors to address:

“*You just need to have a clear evidence base, or some evidence and here's now we're going to do ongoing evaluation… these are the settings that it has evidence for, this is the protocol… That's really important.”* (P18, Manager)

#### Clinical Applications

Clinicians and managers perceived VR to be a “practical” tool with adjunctive applications to existing treatments for disparate mental health disorders. VR was commonly perceived as useful for “exposure work” (*n* = 17) in anxiety disorders, OCD, PTSD, eating disorders, addictions:

“*In vivo exposure has got all sorts of complications and even risks. So minimising risk is fantastic. If the person is anxious about a particular situation that is not easily accessible, at the moment we're relying on imaginal exposure. So, it'll give us another alternative.”* (P19, Manager)

Three clinicians felt VR could be used by patients to practise distress tolerance skills when experiencing self-harm urges (e.g., “*flying through the sky, like a bird… [to] get past their feelings of self-harm”* (P10, Nurse). Other perceived clinical applications included relaxation, mindfulness, functional assessment, psychoeducation, daily living and social skills training, psychodrama, pleasant activities for depression, and preventative training for emergency responders. Notably, staff's views conflicted regarding VR's utility for people experiencing psychotic disorders, with three clinicians expressing concerns:

“*Probably not in conditions like schizophrenia and psychosis. Even in borderline personality disorder there can be times they are micro-psychotic. I'd be concerned of how it would impact on their reality testing”* (P09, Psychiatrist)

Staff also questioned VR's appropriateness for patients with learning and cognitive difficulties (e.g., dementia). Staff perceived benefits in using VR across inpatient, day programs and outreach settings (e.g., increased access, greater control over stimuli), however, questioned its feasibility in group therapy, which was a common delivery mode:

“*Not in the current format we run our groups. We don't have individualised, tailored approaches [which] you would need if you were going to put somebody in that setting.”* (P07, Occupational Therapist)

Some clinicians saw VR offering opportunities to address service gaps within a stepped-care model:

“*I would use it in all OCD patients, if there was an app that they could use at home, on phone and practise any time… I think step after this, would be like people would be able to do it in their own environment, maybe people may not necessarily need to go to see the psychiatrist for early parts if they're not severely impaired.”* (P09, Psychiatrist)

#### Safety and Ethical Concerns

Clinicians' and managers' views varied regarding the clinical; risks of VR. Some perceived no additional risk beyond what was routinely encountered:

“*With the right training about precautions, to use the equipment… doing exposure tasks, I can't really see potential risk issues you wouldn't face ordinarily in day-to-day work.”* (P02, Nurse)

However, nearly half (*n* = 9) expressed concern about patients experiencing adverse effects (e.g., cybersickness, “too distressed”, experiencing dissociation, panic attacks):

“*What that could mean or do in terms of their safety, if they were to have a panic attack as a result of the actual task…also patients who dissociate, feeling out of body, unreal, being in a virtual reality.”* (P04, Psychologist)

Others reflected that the suitability of individuals for VR would depend on illness severity:

“*If the patient [with an eating disorder] is really unwell, it's not suitable for the VR… because the first thing we want [is] to get the… BMI back on track… people with very severe PTSD, having lots of aggression, may damage the VR equipment or maybe… can't be grounded, then it's not going to be helpful.”* (P14, Nurse)

Two clinicians questioned whether VR would interact with psychotropic medications:

“*You've got people who are mentally unwell, on psychotropic drugs. It might affect their interaction with reality at times… I wonder as well… I know for certain antidepressants or certain antimanic drugs, there's that sense of detachment people have… I wonder…. Whether it promotes acceptance or… actually becomes more real. It's a consideration. And whether that might be useful.”* (P13, Nurse)

Others expressed broader ethical concerns including “touching” to prevent patient injury being misconstrued, and harm occurring from inadequate guidelines or monitoring of adverse effects. Thus, staff felt specific protocols would need to be developed to promote safe and ethical usage:

“*There would need to be a really careful assessment stage, or intake process would be a little different… there's a risk… when someone else is developing the content and we just go with it… some of those images could be triggering in other ways.”* (P19, Manager)

### Organisational Factors

Organisational factors were another recurring theme identified from the interviews, which highlighted service contextual factors that may influence the perceived appropriateness and feasibility of VR. This included five subthemes, which identified the importance of having a strong business case, collaborative stakeholder planning, local opinion leaders to champion change, having a service culture supportive of innovation, and understanding resourcing challenges.

#### Business Case

As a private health service, staff perceived a need for therapeutic VR to have clear financial viability. While clinicians perceived management as prioritising “profit margins”, managers described focusing on the additional “value” and sustainability of VR:

“*Hearing how it would benefit our reputation, our brand. There's got to be some business argument, as well as a clinical argument… You won't have people admit themselves into a mental health hospital to do relaxation, but you will [if] they know that we've got cutting edge technology, an intensive program that is going to cut through [their] anxiety disorder. Volume fixes everything* (P19, Manager)

Other considerations identified included proximity to previous quality improvement activities, which could constrain resources or impact organisational stability, and therapeutic VR's alignment with the investment profile of the hospital network's private owners.

#### Collaborative Stakeholder Planning

When implementing new programs, managers identified collaborative planning with stakeholders as key to success:

“*Things fall apart when people haven't taken the time to make a collaborative plan…[with] a voice from… patients, staff, the system, the business structure, and that's a part of the planning process, rather than implementing.”* (P19, Manager)

Staff identified key internal stakeholders (e.g., Medical Advisory Committee) whose guidance and approval would be formally sought, while business stakeholders (e.g., private owners, health funds) were identified as needing more considered engagement:

“*I don't think the health funds have a very intricate knowledge of therapies that we provide. It's not as clear*… *how we treat people… not like a cardiac event… so careful handling of that, because we need our sessions to be funded.”* (P15, Manager)

Managers emphasised the importance of seeking input from all staff “levels” to determine where VR would best fit and to get staff “on board. This was considered especially important given that frontline staff often lacked “*control over outside expectations… put on the service, like health standards and health fund contracts.”* (P17, Manager). To promote awareness and acceptability of therapeutic VR, staff suggested introducing VR at staff meetings and forums and providing opportunities to try VR:

“*The Hospital has the weekly academic forum, which gets quite a cross section of doctors and clinicians, students. The various meetings and committees could be a good place to introduce the concept. Holding an in-service on it and people can have a go.”* (P15, Manager)

Thus, this subtheme highlights the need for collaboration across disciplines and with frontline staff as opposed to implementing VR as a “top down” initiative to implementation.

#### Local Opinion Leaders

Strategic recruitment of staff with formal and informal influence was perceived as important to promote VR uptake, helping establish credibility and maintaining “quality assurance” during its rollout. The support of psychiatrists was considered particularly crucial as a key referral pathway:

“*Our private psychiatrists need to be on board and all working towards the same plan and having that shared understanding so that it is all supported… because they're the ones that admit”* (P15, Manager)

Unit and program managers were also identified as important for promoting acceptability and cultural change within teams:

“*Nurse unit managers–get [them] on board, you realise they're often the ones who have the power. The day programme manager has worked in the hospital for a long time, he's got good rapport with lots of doctors.”* (P16, Manager)

This subtheme, therefore, highlights that opinion leaders need to be recruited from both service leaders and staff at the frontline.

#### Service Culture

Clinicians and managers generally felt VR was a good fit with their service, given its reputation for specialist clinical programs, and as senior management were known for having cultivated a culture for “trialling” new innovations and therapies to maintain broad patient appeal and service competitiveness:

“*They're always open to innovations that help them open to more people coming through, that we're not an organisation that discriminates”*. (P13, Nurse)

“*If there's something they feel… would benefit the patients we have… it's definitely something they will consider… it keeps getting raised that the OCD program is the only… kind in the country. So, anything to keep that momentum.”* (P18, Manager)

Managers described their service as being “consumer-focused”, with decisions to introduce new therapies informed by patient need and feedback:

“*There's been discussion about the day program having some specific mother/baby or parenting groups… That's very much an identified need [from]referrals we're getting, seeing deficit areas people are struggling with.”* (P15, Manager)

This view was shared by clinicians, who emphasised the importance of hearing from patients to ensure VR was “*seen as a valid form of treatment”* (P07, Occupational Therapist).

#### Resourcing Challenges

Clinicians and managers (*n* = 15) unanimously perceived costs associated with purchasing and maintaining VR and providing training to staff as the biggest implementation barrier. However, staff also perceived that “*those barriers [weren't] as bad as if you were to compare to the public system”* (P03, Intake Clinician). One manager noted:

“*If it's part of the inpatient program…we don't get any more money… in some ways… we have a budget for that… like when [we] buy a new car for outreach… it's actually reasonably easy to organise.”* (P16, Manager)

Nonetheless, managers reflected on current resourcing challenges, including “*recruiting appropriately skilled staff* ” (P15, Manager) and rooms, needing further consideration:

“*Space to utilise the technology… You have limited group rooms… [we may] need a room that's purely set up just to do this. If it was a room being used all the time, benefiting a lot of patients, it could be done.”* (P17, Manager)

### Professional Factors

As a broad theme, professional factors focused largely on workforce-related issues that could influence the acceptability and feasibility of VR. This included three sub-themes, highlighting the importance of providing education and training for staff, clinicians' attitudes towards technology and perceptions regarding its usability.

#### Education and Training

Clinicians and managers identified knowledge and skills gaps that needed to be addressed to feel confident implementing VR. Clinicians reported needing training in technical VR skills, assessing patient suitability, and managing ethical and safety risks:

“*The basic safety risks, managing the environment around a person. Understanding the application of it, what the goal is and benefit of it. Being familiar with the scenarios. Potential negative effects. Training in that.”* (P13, Nurse)

Managers reported needing “expert advice” to be informed about the evidence-base, available hardware and software, training resources, and implementation strategies. Suggestions to support staff's educational and training needs included access to treatment manuals, in-service training days, development of clinical governance processes, and consultation opportunities with VR developers and early adopter services.

#### Staff Attitudes Towards Technology

Clinicians and managers (*n* = 16) generally expressed positive attitudes about embracing new technologies. Many perceived broad patient and staff interest in VR given trends “*towards using technology to help us deliver therapies and just in life in general.”* (P12, Psychiatrist). Some clinicians perceived VR as making clinical work easier by saving time organising materials for sessions. One manager noted:

“*We're tending to do more clinical work with [iPads] in terms of apps with clients… We…want… to be able to load them up with documents we use daily… the most common feedback from clinicians is if things can be electronic.”* (P15, Manager)

Whilst participants overall perceived that most staff would be open to the use of VR, they identified that fear of change and resistance to new therapeutic approaches could be barriers for some colleagues. Additionally, a minority were concerned about negative societal impacts of increasing technology use:

“*I think people respond better to the human element. The thought of having machines take over people's care…that organisations might use VR as [a] cost saving way to decrease human labour… there's definitely huge potential harm.”* (P11, Intake Clinician)

“*People are going to be more cut off, ordering food online… starting to get gaming disorders. I'm not saying… suddenly everyone's going to become VR addicts… [but] once we develop something… things [could] get out of hand.”* (P10, Nurse)

Thus, these negative workforce attitudes towards VR were emphasised as needing careful consideration during implementation efforts.

#### VR System Usability

Staff perceived VR to be a relatively simple technology that clinicians and patients could easily learn to use, and were amazed at how “realistic” and “immersive” the VEs were:

“*You're totally transported into that space. You feel quite safe and protected, but just so lifelike, those tendencies coming through, to feel I want to wash the dishes or just wanting to explore.”* (P15, Manager)

However, some felt current VR headsets were "bulky” and “heavy” and raised concerns over patient comfort and practicability in settings without a dedicated set-up (e.g., outreach). Some clinicians perceived their work could be “limited” by software constraints, and anticipated preferencing traditional approaches (i.e., *in vivo* exposure) until “*the technology evolves and gets better”* (P04, Psychologist). Thus, a common suggestion for future application development was for increased customisability:

“*If the therapist could… alter the actual environment, even in small ways, that would be fantastic. Eliminating an object, just to tailor or individualise the setting and treatment.” (*P19, Manager)

Other suggestions to enhance broad clinical utility included developing lighter, more “portable” systems, and greater sensory (e.g., auditory, olfactory, body sensors to “kick” objects) and physiological measurement (e.g., heart rate, perspiration) integration.

## Discussion

This study was the first to explore the perspectives of both clinicians and service managers regarding the enablers and barriers to the implementation of therapeutic VR in mental health services. Three major themes emerged: clinical factors, organisational factors, and professional factors, providing insights into potential barriers and enablers to sustained uptake of VR in the private mental health sector.

### Clinical Factors

The theme, clinical factors, captured perceptions related to clinical appropriateness (24), and confirms VR as having wide applications in mental health services. Enablers included participants' perception of VR as a tool that could enhance patients' engagement with treatment, and strong endorsement for its use in anxiety-related disorders, which is unsurprising given the predominance of exposure-based applications (2). In contrast, uncertainty regarding VR use with people experiencing psychosis and significant cognitive impairment (e.g., dementia) was a barrier, suggesting applications beyond exposure have less intuitive validity and can increase risk perception. Other barriers identified included the mixed views regarding the likely therapeutic efficacy of VR-based interventions, and safety and ethical concerns. These concerns are similar to Lindner et al. (18), in highlighting limited awareness of the current evidence-base, including that of the strong emerging evidence of safe and effective VR applications for individuals living with psychosis and Alzheimer's disease (29–31) and established treatment transfer effects in anxiety disorders (32, 33). This may result in some “pushback” against applications that do not directly replicate current treatment models, which may further limit acceptance of emerging VR applications that “go beyond” what is achievable in real life to address complex, treatment-resistant symptoms (2, 19).

The current findings also suggest that introducing VR may reinforce existing barriers to evidence-based practise uptake, as concerns about patients becoming “distressed” reflect iatrogenic misconceptions that have historically hindered exposure therapy dissemination and led to suboptimal delivery (34, 35). Similarly, CBT for psychosis has achieved variable implementation rates (i.e., 4–100%) and is infrequently offered to patients, which has been attributed to gaps in clinicians' knowledge, inaccurate beliefs regarding patient appropriateness, and prioritisation of pharmacological approaches (36, 37). Thus, addressing provider knowledge gaps and misconceptions will be an important focus of dissemination and implementation efforts (see implications for future implementation).

### Organisational Factors

The theme, organisational factors, emphasised the influence of service context on the perceived appropriateness and feasibility of VR (24). While financial, staffing, and logistical resourcing challenges were perceived to be the greatest barrier, the facilitatory role of a strong business case in an organisation's decision to adopt VR was highlighted. Notably, considerations for economic viability tend to be more salient for private organisations (38). As therapeutic VR is not currently reimbursed by private health funds, services may lack sufficient incentive to adopt VR if it is more costly than current practises. Thus, in making a business case for VR, it may be helpful to consider Rogers' Theory of Innovation Diffusion (39), which highlights the influence of innovation attributes on implementation outcomes. Providing staff opportunities to trial therapeutic VR environments and emphasising its “relative advantages” over current practises and workflows will likely be beneficial. For instance, VR can overcome challenges associated with delivering exposure therapy (e.g., inaccessible stimuli) and there is evidence to suggest it can be more acceptable to patients (40, 41), which may increase service demand and engagement.

Interestingly, research suggests that mental health services in the private compared to public sector, are more likely to provide support for new evidence-based practise (e.g., resource allocation, training, supervision), leading to more positive provider attitudes towards their use (21, 42). This was reflected in the current data, which identified a service culture that valued patient-centred care and innovation. The influence of service culture was evident in staff's overall positive attitudes for therapeutic VR, which appeared reinforced by historic organisational interest and support for adopting new therapies.

Importantly, collaborative engagement with key clinical and business stakeholders prior to, and during implementation, was emphasised as critical to understanding the complexities of change across system levels and to maximise adaptation of VR to the service context. This would further allow strategic recruitment of local opinion leaders as “change agents” (43) as studies have identified colleague endorsement and expertise in VR as facilitators to improved provider acceptance and intentions to adopt VR in neurorehabilitation settings (44, 45). For instance, middle management and “first-level leaders” have been identified as having key roles in influencing staff attitudes and acceptance of evidence-based practises, given their bridging function with senior leadership and proximity to frontline staff (46, 47).

### Professional Factors

The theme, professional factors, highlighted perceived acceptability and feasibility issues at the workforce level. Specifically, concerns about VR “replacing jobs” were identified as a potential barrier. These reflect similar concerns identified in the VR neurorehabilitation literature (48, 49) and speak to the disruptive nature of technologies, which in helping patients build capacity to “self-manage” their care, can also “threaten” current practise models (50, 51). The emergence of automated applications, which may be delivered by a “non-specialist workforce” providing predominantly technical support (52) will likely enhance these concerns.

Perceived usability issues of VR systems, including the “heaviness” and lack of portability of headsets and limited customisability of environments, were also raised as a barrier. This is particularly notable as they, to a large extent, reflect limited awareness of the current state of technology. For instance, affordable, lighter, standalone headsets (e.g., Oculus Quest 2, Pico Goblin, HTC Focus), and customisable therapeutic software (25) are rapidly becoming industry standard, while anticipated technological advancements in full-body haptics and multi-user systems promise to offer greater immersion and opportunities for use in group therapy settings.

A need for development and dissemination of quality educational and training resources emerged as a critical requirement to address the limited awareness of the current state of clinical evidence and VR technology. Indeed, innovations are more easily adopted when their required procedural knowledge is codified and when access to high-quality training and fidelity-based supervision is available (43, 53). Clarifying the role of VR within the clinical workflow, the ongoing need for clinical skill (e.g., assessment, psychoeducation, monitoring) in providing access to VR-based interventions, as well as the potential consumer benefits, may also alleviate identified workforce concerns by reinforcing professional values, including altruistic motivations to help patients (21, 54, 55).

### Implications for Future Implementation

According to Proctor et al.'s (24) highly influential framework of implementation outcomes, in the early stages of implementation (i.e., where therapeutic VR for mental health care sits currently) understanding perceived acceptability, appropriateness and feasibility are crucial to aid adoption and sustained uptake. These were clearly reflected in the current findings, and have practical implications for future dissemination and implementation efforts. Limited telehealth use prior to the COVID-19 pandemic demonstrates that technology accessibility does not guarantee successful dissemination if providers remain uninformed about evidence-based practises or perceive potential risks as outweighing benefits (56). Thus, given the lack of specific clinical guidelines, and limited treatment protocols and training programs for therapeutic VR, their development and dissemination should be prioritised. Such resources will be critical to enhance provider confidence and competence to select appropriate VR systems for their service and patients' needs, particularly considering the rapid pace that VR technology and its therapeutic evidence-base is advancing. Consultation opportunities with early adopters of VR-based therapies may promote uptake by improving providers' perceived capacity to manage implementation risks (21, 57). Targeting concerns and misconceptions related to therapeutic VR's clinical appropriateness will be critical given the stronger influence of negative attitudes (negative predictor) than positive on therapists' intention to use it (57). It would also be beneficial to embed VR into clinical training programs as part of developing competencies in digital mental health (56). As the current data were also similar to constructs common in theoretical frameworks [e.g., Consolidated Framework for Implementation Research (58), Theoretical Domains Framework (59)], these may be suitable tools to guide comprehensive identification of mechanisms of change targets and development of relevant implementation strategies to enhance uptake of therapeutic VR.

The perspectives documented highlighted additional under-researched areas warranting investigation. Given the ubiquitous use of psychotropic drugs in psychiatry, their impacts on VR immersion remains a significant practise issue. The extant literature suggests common pharmacological drugs (e.g., antidepressants, antipsychotics) have minimal influence on concurrent VR usage (30, 60–63), however, higher attrition rates and PTSD symptoms relative to controls have been reported from VR interventions in conjunction with alprazolam and dexamethasone (61, 64). This will be particularly relevant with the rise of psychedelic pharmacotherapy for psychiatric indications (65), as the combination could conceivably help or hinder therapeutic outcomes. Additionally, our understanding as to which patients are “good candidates” for VR therapies, the effect of combined treatment, and the optimal number and frequency of sessions is limited.

### Strengths, Limitations, and Future Directions

The current study has notable strengths in being the first to recruit cross-disciplinary mental health clinicians and managers across organisational levels, using semi-structured questions to allow naturalistic themes to emerge, providing staff an opportunity to experience a therapeutic VR, and in exploring structural barriers to implementation. The findings, should be considered with some limitations in mind, including its specificity to private sector health services and the higher proportion of nursing staff in the sample, relative to other clinical disciplines (psychiatrists, psychologists, allied health). This does, however, reflect the typical staff distribution within mental health hospitals. Moreover, with emerging evidence for automated applications (e.g., virtual therapy coach), VR is increasingly likely to be delivered by any clinicians irrespective of their training background, whose role would be primarily to provide a technical or supportive role (66). Going forward, expanding findings to public mental health services will be valuable. It will be especially important to investigate the perspectives and needs of consumers of mental health services, and to communicate these to service providers and embed them in implementation initiatives. Future research may consider applying broader theoretical frameworks to guide identification of behavioural determinants and implementation intervention development.

## Conclusion

The implementation of therapeutic VR for mental health has potential to enhance clinical care and patient engagement with treatment. A rapidly growing body of high-quality evidence supports its efficacy for treating a range of mental illnesses, and recent commercial availability of high-quality VR headsets have greatly aided early implementation efforts. However, adoption of new evidence-based practises can take decades, with implementation of technology-based innovations even more challenging. Despite concerns about resourcing and feasibility across different settings, this study identifies enablers for VR adoption in private mental health settings including promotion of service innovation cultures, development of a strong business case, education and training opportunities, stakeholder engagement and recruitment of opinion leaders to maximise service implementation. Key foci for future dissemination include developing evidence-based clinical guidelines, training resources and implementation interventions to promote its safe, ethical, and sustainable use.

## Data Availability Statement

The raw data supporting the conclusions of this article will be made available by the authors, without undue reservation.

## Ethics Statement

The studies involving human participants were reviewed and approved by Melbourne Clinic Human Research Ethics Committee (#304) and the Monash University Human Research Ethics Committee (#13284). The patients/participants provided their written informed consent to participate in this study.

## Author Contributions

RS, TR, OC, and AJ were involved in initial study conceptualisation. OC was responsible for data collection, data analysis, and manuscript drafting. AJ contributed to data collection. RS and TR contributed to data analysis. OC, RS, ND, TR, MY, and CN critically reviewed the data interpretations and made significant contributions to the final manuscript version. All authors approved of the submitted version.

## Funding

This research was supported by an Australian Government Research Training Program Scholarship and the David Winston Turner Endowment Fund. The funding sources had no role in the design, management, data analysis, presentation, interpretation, and write-up of the data.

## Conflict of Interest

The authors declare that the research was conducted in the absence of any commercial or financial relationships that could be construed as a potential conflict of interest.

## Publisher's Note

All claims expressed in this article are solely those of the authors and do not necessarily represent those of their affiliated organizations, or those of the publisher, the editors and the reviewers. Any product that may be evaluated in this article, or claim that may be made by its manufacturer, is not guaranteed or endorsed by the publisher.
