# Corrigendum: Implementation of Therapeutic Virtual Reality Into Psychiatric Care: Clinicians' and Service Managers' Perspectives

**DOI:** 10.3389/fpsyt.2022.893637

**Published:** 2022-06-23

**Authors:** Olivia S. Chung, Tracy Robinson, Alisha M. Johnson, Nathan L. Dowling, Chee H. Ng, Murat Yücel, Rebecca A. Segrave

**Affiliations:** ^1^BrainPark, Turner Institute for Brain and Mental Health and Monash Biomedical Imaging Facility, Monash University, Melbourne, VIC, Australia; ^2^School of Nursing, Paramedicine and Healthcare Sciences, Charles Sturt University, Bathurst, NSW, Australia; ^3^Professorial Unit, Department of Psychiatry, The Melbourne Clinic, The University of Melbourne, Melbourne, VIC, Australia

**Keywords:** implementation, virtual reality, barriers, facilitators, qualitative study, mental health, psychiatry

In the original article, under the “Organizational Factors” and “Professional Factors” sections of the Discussion, the citation numbers from #39 onwards were listed incorrectly. Additionally, two references were missing from the original reference list:

Levac D, Glegg SMN, Sveistrup H, Colquhoun H, Miller PA, Finestone H, DePaul V, Harris JE, Velikonja D. A knowledge translation intervention to enhance clinical application of a virtual reality system in stroke rehabilitation. *BMC Health Serv Res*. (2016) 16:557. doi: 10.1186/s12913-016-1807-6

Threapleton K, Newberry K, Sutton G, Worthington E, Drummond A. Virtually home: feasibility study and pilot randomised controlled trial of a virtual reality intervention to support patient discharge after stroke. *Br J Occup Ther*. (2018) 81:196–206. doi: 10.1177/0308022617743459

The reference list has now been corrected (new references 44 and 48 signified by ^***^).

In the “Discussion” section, under “Professional Factors” “The emergence of automated applications, which may be delivered by a ‘non-specialist workforce' providing predominantly technical support (45) will likely enhance these concerns” has been corrected to:

“The emergence of automated applications, which may be delivered by a “non-specialist workforce” providing predominantly technical support (52) will likely enhance these concerns.”

“Indeed, innovations are more easily adopted when their required procedural knowledge is codified and when access to high-quality training and fidelity-based supervision is available (43, 52)” has been corrected to:

“Indeed, innovations are more easily adopted when their required procedural knowledge is codified and when access to high-quality training and fidelity-based supervision is available (43, 53).”

“Clarifying the role of VR within the clinical workflow, the ongoing need for clinical skill (e.g., assessment, psychoeducation, monitoring) in providing access to VR-based interventions, as well as the potential consumer benefits, may also alleviate identified workforce concerns by reinforcing professional values, including altruistic motivations to help patients (21, 47, 53)” has been corrected to:

“Clarifying the role of VR within the clinical workflow, the ongoing need for clinical skill (e.g., assessment, psychoeducation, monitoring) in providing access to VR-based interventions, as well as the potential consumer benefits, may also alleviate identified workforce concerns by reinforcing professional values, including altruistic motivations to help patients (21, 54, 55).”

In the **Discussion** section, under “Implications for Future Implementation,” “Limited telehealth use prior to the COVID-19 pandemic demonstrates that technology accessibility does not guarantee successful dissemination if providers remain uninformed about evidence-based practises or perceive potential risks as outweighing benefits (54)” has been corrected to:

“Limited telehealth use prior to the COVID-19 pandemic demonstrates that technology accessibility does not guarantee successful dissemination if providers remain uninformed about evidence-based practices or perceive potential risks as outweighing benefits (56).”

“Consultation opportunities with early adopters of VR-based therapies may promote uptake by improving providers' perceived capacity to manage implementation risks (21, 55)” has been corrected to:

“Consultation opportunities with early adopters of VR-based therapies may promote uptake by improving providers' perceived capacity to manage implementation risks (21, 57).”

“It would also be beneficial to embed VR into clinical training programs as part of developing competencies in digital mental health (54). As the current data were also similar to constructs common in theoretical frameworks [e.g., Consolidated Framework for Implementation Research (56), Theoretical Domains Framework (57)], these may be suitable tools to guide comprehensive identification of mechanisms of change targets and development of relevant implementation strategies to enhance uptake of therapeutic VR” has been corrected to:

“It would also be beneficial to embed VR into clinical training programs as part of developing competencies in digital mental health (56). As the current data were also similar to constructs common in theoretical frameworks [e.g., Consolidated Framework for Implementation Research (58), Theoretical Domains Framework (59)], these may be suitable tools to guide comprehensive identification of mechanisms of change targets and development of relevant implementation strategies to enhance uptake of therapeutic VR.”

“The extant literature suggests common pharmacological drugs (e.g., antidepressants, antipsychotics) have minimal influence on concurrent VR usage (30, 58–61), however, higher attrition rates and PTSD symptoms relative to controls have been reported from VR interventions in conjunction with alprazolam and dexamethasone (59, 62). This will be particularly relevant with the rise of psychedelic pharmacotherapy for psychiatric indications (63), as the combination could conceivably help or hinder therapeutic outcomes. Additionally, our understanding as to which patients are “good candidates” for VR therapies, the effect of combined treatment, and the optimal number and frequency of sessions is limited” has been corrected to:

“The extant literature suggests common pharmacological drugs (e.g., antidepressants, antipsychotics) have minimal influence on concurrent VR usage (30, 60–63), however, higher attrition rates and PTSD symptoms relative to controls have been reported from VR interventions in conjunction with alprazolam and dexamethasone (61, 64). This will be particularly relevant with the rise of psychedelic pharmacotherapy for psychiatric indications (65), as the combination could conceivably help or hinder therapeutic outcomes. Additionally, our understanding as to which patients are “good candidates” for VR therapies, the effect of combined treatment, and the optimal number and frequency of sessions is limited.”

In the **Discussion** section, under “Strengths, Limitations, and Future Directions,” ‘Moreover, with emerging evidence for automated applications (e.g., virtual therapy coach), VR is increasingly likely to be delivered by any clinicians irrespective of their training background, whose role would be primarily to provide a technical or supportive role (64)” has been corrected to:

“Moreover, with emerging evidence for automated applications (e.g., virtual therapy coach), VR is increasingly likely to be delivered by any clinicians irrespective of their training background, whose role would be primarily to provide a technical or supportive role (66).”

The authors apologize for these errors and state that this does not change the scientific conclusions of the article in any way. The original article has been updated.

## Publisher's Note

All claims expressed in this article are solely those of the authors and do not necessarily represent those of their affiliated organizations, or those of the publisher, the editors and the reviewers. Any product that may be evaluated in this article, or claim that may be made by its manufacturer, is not guaranteed or endorsed by the publisher.
