# Supplementary material for: Implementation of Therapeutic Virtual Reality Into Psychiatric Care: Clinicians' and Service Managers' Perspectives
Source: Front Psychiatry. 2022 Jan 4;12:791123. doi: 10.3389/fpsyt.2021.791123 (PMC8764380; doi:10.3389/fpsyt.2021.791123)
Supplement: Supplementary file 2 [file Table_2.docx]

Supplementary Item S2

## Table 3. Illustrative quotations by theme

| **Themes/subthemes** | **Quotations** |
| --- | --- |
| ***Clinical factors*** | |
| Patient engagement | *“It could be viewed as a less threatening way of getting treatment. It could be less anxiety provoking. For example, for someone with OCD, if they know the treatment involves exposure to - repeated exposure to the real thing around what their issues are VR might be seen as something that is a gentler introduction to it. The same with anxiety too, I would imagine. So if you had someone who was afraid of flying, VR would be a very practical way of dealing with the anxiety as opposed to actually going on a plane”* (P02, Nurse)  *“Sometimes having a new modality can be the one thing that might help you to break through with a patient if other things have failed [or] who might be stuck in a bit of a rut with just using verbal talking therapies as a way to try and overcome a problem... Severe PTSD patients become very avoidant because the stimulus is so unpleasant. They may be open to doing things in a small, controlled way that might enable treatment to happen earlier.”* (P09, Psychiatrist)  *“With the younger generation, technology is a given now. It could be a segue into their world of interaction… Let's take, hardcore gamers… Here's a situation where you've got something they can buy into… It could be a nice way of introducing therapeutic concepts… It might still appeal to those older people, depending how we couch to them. It's about getting people on board with new advances, away from pharmaceuticals often. You have those two opposing [views] where there are people who think pharmaceuticals are the be all and end all, this is what's going to cure them, to those that absolutely don’t want to touch them at all.”* (P13, Nurse)  *“I used to run relaxation sessions, breath work, muscle relaxation, imaginary stuff. Probably 20% of people said they can't imagine a scene in their mind. With this you wouldn’t have to imagine it.”* (P07, Occupational Therapist)  *“It’s probably a new subset that we maybe haven’t seen as much of or haven’t found some of the models of treatment as helpful. Being immersed in something like that, being very visual, it could really open doors for people that may not have followed through with treatment.”* (P03, Nurse)  *“I think it could be used to help people who find it very difficult to be in group situations [therapy] and who want to just to talk to someone. It might be a great way - a great tool to break down some barriers for some people.”* (P10, Nurse) |
| Therapeutic efficacy | *“[There’s] a pretty high bar for evidence-based treatments. It might struggle to be taken up if there's not as much evidence for it, or in competition with other more established therapies. Then there's the question of therapeutic drift. If someone's running an ACT program and they had VR on the side, would that compromise?”* (P06, Occupational Therapist)  *“You just need to have a clear evidence base, or some evidence and here's now we're going to do ongoing evaluation… these are the settings that it has evidence for, this is the protocol… That's really important… Usually, it's taken to the Medical Advisory Committee for the senior psychiatrist to look over the evidence supporting it. Generally, they're quite happy if something's been tested overseas.”* (P19, Manager)  *“Having an evidence-base, that's drilled into you as a clinician. I think that would overcome a lot of the barriers to cost and training* (P04, Psychologist)  *“Whether [VR] would translate to the same kind of clinical results… something tells me that in vivo would increase anxiety levels more than the VR, which would mean it would probably have better outcomes.” (Psychologist 1, female, 20s)*  *“I can see it being useful in terms of yeah, OCD and exposure. I guess it would be interesting to see the translation between the feeling, touching things imagined, with not the real hand and then actually doing that for real.”* (P09, Psychologist) |
| Clinical applications | *“In vivo exposure has got all sorts of complications and even risks. So minimising risk is fantastic. If the person is anxious about a particular situation that is not easily accessible, at the moment we're relying on imaginal exposure. So it'll give us another alternative.”* (P19, Manager)  *“I'd actually love to use it for anxiety or even social anxiety that they could even go into that and practice conversations with people or going up to people. I could imagine that being helpful. Whether they can get people to talk back, I don't know… a lot of people you find need a lot of help with communication skills.”* (P08, Psychologist)  *“It would be amazing to have at the Day Program, when people are choosing to use distress tolerance skills because they've been activated. We spend a lot of time using cold water and other sensory tools. But our space is limited and often it's a busy kitchen environment... if you could have three of those hanging out at the Day Program, people could whip them on as a self-guided chill out zone, meditation time.”* (P07, Occupational Therapist)  *“Potentially you could be using it with people with PTSD, but not in the current format we run our groups. You'd need individual workers to establish where that's appropriate. My other thought is an inpatient setting might make more sense, because you don't have access to lots of environments, whereas people going home that night, they've got access to the world.”* (P07, Occupational Therapist)  *“I think it can be used for body dysmorphic disorders…because I think one of the things we notice is there's such a discrepancy between their own body shape as well as their perception. So I was thinking there need to be some way of virtual reality thing that we could do to actually expose them into the proper body shape.”* (P09, Psychiatrist)  *Probably not in conditions like schizophrenia and psychosis. Even in borderline personality disorder there can be times they are micro-psychotic. I’d be concerned of how it would impact on their reality testing… I probably would use it in all OCD patients, if there was an app that they could use at home, on the phone and practise any time... I think step after this, people [will] not necessarily need to go to see the psychiatrist for early parts, if they're not severely impaired”* (P09, Psychiatrist)  *“People with psychosis–I can't think of any scenarios you'd use. Where the connection with reality is already tenuous. Challenging that is going to be counterproductive.”* (P13, Nurse)  *“Paranoid delusional type disorders could have applications… This is something I know has already been developed… they were in an elevator, there were some people in there and their eye gaze would shift. I could see how that would be really uncomfortable and actually really good exposure work for somebody with those kinds of delusions.”* (P04, Psychologist)  *“Maybe if they somehow could benefit from maybe not cutting but doing something else… Something that's so totally relaxing and amazing - they fly like a bird, going through canyons and… for that period of time you just - you're in another world, and it's a beautiful world, and it's quite exciting and maybe they'll get past their feelings of self-harm.”* (P10, Nurse)  “*Looking at this technology to give people wonderful experiences, of trying to create, okay, you want a wonderful life, what's your idea? How would your day look if you didn't feel depressed anymore?... Somehow being able to immerse somebody in that kind of experience, so they can then have a picture and things in their mind of, okay, this is - yeah, this would be what that might look like to me. I think that would help to go a long way to creating hope for people*… *when you visualise things that changes how you feel.*” (P11, Intake Clinician) |
| Safety and ethical concerns | *“With the right training about precautions, to use the equipment… doing exposure tasks, I can't really see potential risk issues you wouldn't face ordinarily in day-to-day work.”* (P02, Nurse*)*  *“It could be that if there's certain risk identified in some client groups, we are doing it here where we've got the support of the hospital or clinical staff, or in the psychiatrist's rooms for the first time…[but] I think as fairly seasoned clinician, you'd feel comfortable to trial it and if there was a reaction that was negative or upsetting… manage that just as you would with anything else.”* (P15, Manager)  *“What that could mean or do in terms of their safety, iIf they were to have a panic attack as a result of the actual task with the headset on… also patients who dissociate­, feeling out of body, unreal, but being in a virtual reality, I think that would be really confusing and maybe pretty confronting. I'd probably be concerned about that.”* (P04, Psychologist)  *“There would need to be a really careful assessment stage, or intake process…given the nature of immersing yourself in a different space…There's a risk when someone else is developing the content…could be triggering in other ways…A way to mitigate that risk might be [showing] pictures of the environment and [making] sure the person feels comfortable.”* (P19, Manager)  *“Like anything new that's used, there are always those unforeseen elements, especially when you're dealing something that really is distorting somebody's perception of reality, even on a minimal level. Do you know what's going to occur? You've got people who are mentally unwell. On psychotropic drugs it might affect their interaction with reality at times. Maybe that's just a thought of mine.”* (P13, Nurse)  *“Maintaining safety–wanting to physically intervene if something has happened, but there are also ethical risks involved in touching a person without permission, especially as a male clinician.”* (P13, Nurse)  *“Concerns in the hierarchy about proof beforehand that it works well, works properly, doesn't make people worse, those kinds of things. They might want cast iron guarantees that this won't make people worse, even if it doesn't make them better.”* (P07, Occupational Therapist)  *“People with severe PTSD, having lots of aggression, may damage VR equipment or [if they] can’t be grounded, then it’s probably not going to be helpful. It will cause a lot of stress, even for the clinical staff conducting it.”* (P14, Nurse)  *“My only concern, it seems like every time a new treatment is developed in psychiatry, everyone gets it… when ECT first came out, when chlorpromazine came out, everyone got it. It's like we grab a new technology, and we throw it out there to everyone, because we want to fix as many people as possible…everyone falls into that trap and looking back in history, there doesn’t seem to be anyone who's watching it. I guess researching the effects of it and trying to monitor [it would be important] and these researchers have nothing to do with it, don't provide virtual reality.”* (P10, Nurse) |
| ***Organisational factors*** | |
| Business case | *“I guess it would have to be some sort of cost benefit to the normal clinic. I don't know whether that means that there's going to be this whole new group of potential patients who are going to come to a hospital for a virtual reality program. Like, wow, yes, I've heard that you're doing virtual reality OCD treatment. Then these people - there might be a whole bunch of people that suddenly in the future will be coming just for that.”* (P10, Nurse)  *“It's a private hospital, private comes first. But that's not totally true. That's - no. I think if… whoever was doing it could demonstrate the benefits at a reasonable cost.”* (P11, Intake Clinician)  *“They'll have to see a profit margin. It would have to demonstrate a certain efficacy before they'd think about doing it. But implementing might be different if it's costly*… *Even promoting the fact if we do it here… this is a pilot site. That's a hell of a lot of kudos to the place. Hey, we did this first. We got our name on this. Bit of pride. As an organisation… I think it's putting in a way that makes it - hey, this will be good for us not just financially but also reputationally. That would be important* (P13, Nurse)  *“When a patient is an inpatient, we don't get any more money for doing extra, like doing TMS. But… if you thought it was going to make a big difference in the life of somebody… we would just do it. We do things that are labour intensive because they add value for the patient.”* (P16, Manager)  *“Hearing how it would benefit our reputation, our brand. There's got to be some business argument, as well as a clinical argument… There's barriers to exposure. If we're saying, we'll use it for exposure therapy, it will draw or keep patients in, it's effective, we know it works; then they'll give you the money. We use it for relaxation, it's not a priority anymore because you won't have people admit themselves into a mental health hospital to do relaxation, but you will have people admit themselves because they know that we've got cutting edge technology and an intensive program that is going to cut through your anxiety disorder. Volume fixes everything.”* (P19, Manager) |
| Collaborative stakeholder planning | *“Collaboration with stakeholders is really key to success–there's a voice from the perspective of patients, staff, the system, business structure, and that's a part of the planning process, rather than implementing.”* (P19, Manager)  *“It needs to be a collaborative process. We don’t have a lot of control over outside expectations put on the service, like health standards and health fund contracts. Getting the team engaged in change is the best way of going about it.”* (P17, Manager)  *“The psychiatrists are always going to be the group that have to be very carefully handled, and the health funds to some degree. The other consideration with all of our treatments here is that the health funds are on board with it being a current evidence-based therapy tool... I don't think [they] have a very intricate knowledge of therapies that we provide. It’s not as clear… how we treat people, not like a cardiac event, or a neurological disturbance. So careful handling of that because we need our sessions to be funded.”* (P15, Manager)  *"Getting clinicians to have a go, that's actually really helpful because people can shut off or turn down things that they actually haven't experienced or are afraid to experience themselves.”* (P04, Psychologist)  *“In terms of the personal barriers people have, getting them familiar with the technology, getting the buy-in from them, saying, this is cool, it will work, something interesting to try.. Exposing them to it. Getting people excited about it.”* (P13, Nurse)  *“Definitely staff experiencing it too. The Hospital has the weekly academic forum which gets quite a cross section of doctors and clinicians, students. The various meetings and committees could be a good place to introduce the concept. Holding an in-service on it and people can have a go.”* (P15, Manager) |
| Local opinion leaders | *“The manager might have to tell the nursing staff, you have to go and do this for the psychologist. Oh but I can't because I don't know anything about it, that's what some nurses might say… I think maybe from them, like if they say well we've done our sample, it's good, this is how I felt, and then I think everyone else will say, okay so she's tried it, or he's tried it, and it's okay. So we'll try it.”* (P01, Nurse)  *“Getting the doctors on board with referring for [VR]. Generally, our work is generated from doctors making referrals.”* (P17, Manager)  *“I’d imagine, getting some key doctors on board would be really helpful and having doctors refer to a program, or whether it’s a research endeavour to begin with.”* (P03, Nurse)  *“Having a lot of the doctors on board. Taking it to lunch time meetings where it could be discussed, getting the psychiatrists to try it.” (P05, Counsellor)*  *“I think all of, or as many of, our private psychiatrists need to be on board and all working towards the same plan and having that shared understanding so that it is all supported…once it's been approved… because they're the ones that admit”* (P15 Manager)  *“Nurse unit managers–get [them] on board, you realise they're often the ones who have the power. The day programme manager has worked in the hospital for a long time, he's got good rapport with lots of doctors. Somebody like him trialling this would be great too.”* (P16, Manager) |
| Service culture | *“The Melbourne Clinic, especially the rainbow tick they got recently… Looking at the diverse community and being open to therapies that don’t discriminate. They're always open to those kinds of innovations or things that help them open to more people coming through, that we're not an organisation that discriminates.”* (P13, Nurse*)*  *“There's been discussion about the day program having some specific mother/baby or parenting groups. We don't have that at the moment. That's very much an identified need [from] the types of referrals we're getting and seeing the deficit areas people are struggling with.”* (P15, Manager)  *“EMDR started as an individual thing and has evolved to be a group program to meet the needs of clients. We're quite good at identifying a need and adapt[ing] to feedback.* (P17, Manager)  “*The Melbourne Clinic I also find is big on consumer advocacy and really consumer focused in the past couple of years, and if there's something that they feel as though it would benefit the patients that we have through the door then it's definitely something that they will consider. I think it's always being considered, the staff - the management team that we've got there at the minute are really kind of like forward thinking and always looking to implement change anyway and not to stay stagnant. I think it keeps getting raised that the OCD programme is the only programme of its kind in the country anyway. So, anything to keep that momentum for the programme.”* (P18, Manager)  *“There could be three different ways a client could be getting treatment. Ultimately, it's up to that client to decide. Having another delivery system or modality to work within… It’s not far from what we currently do… Those choices have to be based [on] clinical reasoning. But as long as they are, sometimes we can't predict which one will be best.”* (P07, Occupational Therapist) |
| Resourcing challenges | *“It’s always money. Space. Having rooms available. I think in a private facility, those barriers aren’t really as bad as say if you were to compare to the public system"* (P03, Intake Clinician)  *“Well the cost I think would be a barrier. I don't know how much the units cost, I don't know much the software costs, but I think that would probably be a barrier for most clinics. The initial setup of those things - like that thing's giant and it's not a regular - it doesn't look like a regular computer so there would be costs associated. Space - so having a room that is available that has nothing in it. Yeah, they're the things coming to mind. I guess also making sure that everybody's trained so that takes time, effort, money, all of those things to be able to use the system too.”* (P04, Psychologist)  *“Straight up the HR issues of the skill mix, recruiting appropriately skilled staff… capital expenditure. It may need to be a standalone space. Anything we're trying to implement, it's the right set up, money is invested in it, doctors have to be on board. It's never easy. Although we managed to upgrade all the iPhones and purchase 11 new fleet [cars].”* (P15, Manager)  *“Space to utilise the technology. The technology itself, access. Costs. You have limited group rooms... If it was a room being used all the time, benefiting a lot of patients, it could be done.”* (P17, Manager)  *“We seem to be a bit behind in technology here. I can't see it happening any time soon. Probably that mostly. Because I think there's no reason we couldn't do that [introduce VR].”* (P08, Psychologist) |
| **Professional factors** |  |
| Education and training | *“It would need to have a really clear guide as to where it's going to most likely have effectiveness. Information and education about how to assess for that and put people in.”* (P07, Occupational Therapist).  *“If I get more training I'll be [confident]. Maybe a one-day course explaining the basics. Like with CBT. A manual would be nice.* (P09, Psychiatrist)  *“The basic safety risks, managing the environment around a person. Understanding the application of it, what the goal is and benefit of it. Being familiar with the scenarios. Potential negative effects. Training in that.”* (P13, Nurse)  *“Consultation [with VR developers]. Where else is it being used or trialled? That would be really good for us to immerse in that, just to be as informed and knowledgeable… [Knowing how to] evaluate the impact–cost and clinically… We would need a lot of expert advice. But we would be very keen.”* (P15, Manager) |
| Staff attitude towards technology | *“These days we live in a very technological age with phones and a lot of visual stimulation… things are moving very much towards using technology to help us deliver therapies and just in life in general. So I think there would be interest in it.”* (P12, Psychiatrist)  *“From the clinician point of view just the excitement of something completely different, whilst you've still got that relationship and rapport. It's taking some of the pressure off you. A lot of what we do is talking our clients through things. You'd still be doing that but so enhanced to have at your fingertips other material [to create an] experience that can be difficult to elicit depending on where people are at with their illness and symptoms… [We’re] tending to do a lot of more clinical work with them [iPads] in terms of apps with the clients, presentations for our team days in. We really…want to push for the IT and the CapEx commitment to be able to load them up with documents that we use daily… that's probably the most common feedback from the clinicians is that if things can be electronic.”* (P15, Manager)  *“I think people respond better to that human element. The thought of having machines take over people's care…that organisations might use virtual reality as some kind of cost saving way to decrease human labour…there's definitely huge potential harm in that.”* (P11, Intake Clinician)  *“At first, I was sceptical, because I feel like the whole world is becoming more technological, people are going to be more cut off, ordering food online… all the time now, on mobile phones…starting to get to gaming disorders. I'm not saying… suddenly everyone's going to become virtual reality addicts… [but] I think once we develop something… things [could] get out of hand.”* (P10, Nurse |
| VR system usability | *“Right now, I think it would be difficult simply because of the bulkiness of the equipment, but in future maybe when there's no need for the sensors… if you could have community clinicians doing exposure tasks in an outreach setting, then that's going to be hugely beneficial… Training wise, I think the average person, if you can use a computer you can set up VR, so I don't really see that as a barrier either. Community yes, the equipment is still a bit bulky. It would be difficult to do it in the community setting as the equipment is at the moment, but I suspect that will change and evolve as time goes on.”* (P02, Nurse)  *“It's really quite heavy at the front so I can't imagine patients would be wanting to wear it for too long. It's definitely effective, but I think just the headset is a bit cumbersome… I [still] think it's the right time for therapists to start using this sort of technology. It's much smaller. It's still expensive, but more affordable than it used to be… If the therapist could change the program, alter the actual environment, even in small ways, that would be fantastic. Eliminating an object, just to tailor or individualise the setting and treatment.”* (P19, Manager)  *“With a portable modality you could bring the modality to the patient and they could be practising it in their own time, their own space. For someone who's very impaired, like someone with severe OCD, who's barely getting out of the house… that could really enhance things. Getting some of those guys to come along to appointments can be really difficult.”* (P09, Psychiatrist)  *“If you could somehow tailor the virtual reality to that person's situation, so it's not just something that you just give to all people with OCD… but maybe you could… almost produce a virtual reality film, whatever you call them… that somehow really relates [to their issues].”* (P10, Nurse)  *“If it was something you could do in reality, I'd probably choose that over the VR at this stage. But as the technology evolves and gets better that will probably eliminate that.”* (P04, Psychologist)  *“I don’t think it's really highly complicated, that would probably exclude potential users from it… But I think it's in its very infancy and it would need to be developed a lot more, even though there's a visual thing… to make it more on par with reality, it would probably need to have more of the sound.””* (P11, Intake Clinician)  *“I'm pretty sure everyone can learn it within 10 minutes because my little daughter, three years old, can play it. It's very, very easily learnt, you just have to give them a sample, just show them what they're supposed to do.”* (P01, Nurse)  *“If it was something that you could do in reality I'd probably choose that over the VR at this stage. But as the technology evolves and gets better that will probably eliminate that”* (P04, Psychologist)  *“Maybe you can do more monitoring and to see like the heart beat or different perspiration. People can understand how we [are] actually affecting why we feel palpitation, why their heart beats go up and things.”* (P14, Nurse) |
